# Standing chest compressions: simulation-based development and evaluation of a clinically applicable height adjustment approach

**DOI:** 10.1016/j.resplu.2026.101479

**Published:** 2026-09-04

**Authors:** Alisha Izadi, Selina Kim, Marc Lazarovici, Stephan Prückner, Heiko Trentzsch

**Affiliations:** Institut für Notfallmedizin und Medizinmanagement, LMU Klinikum, Ludwig-Maximilians-Universität München, Schillerstrasse 53, 80336 Munich, Germany

**Keywords:** Cardiopulmonary resuscitation, Closed heart massage, Workplace ergonomics, Posture, Cardiac arrest, Manikin

## Abstract

**Introduction:**

When hospital staff perform cardiopulmonary resuscitation, patients are usually lying on a bed or stretcher, and chest compressions are therefore commonly performed while standing. Current resuscitation guidelines do not provide recommendations regarding standing chest compressions. The aim of this study was to identify a favourable working height for high-quality standing chest compressions.

**Material and methods:**

This prospective study included ten healthcare professionals and twenty medical students and was conducted in two parts. Firstly, chest compressions were performed at seven different working heights, starting from knee height as a reference mark. Additional heights were set at 10, 15, and 20 cm above (+) and below (−) knee height. Secondly, knee height setting was then evaluated by comparing chest compression quality with two clinically relevant reference conditions: kneeling on the floor and standing at the lowest stretcher height.

**Results:**

In Part 1, the highest values for the primary outcome measures – mean compression depth (52.7 ± 7.9 mm) and the percentage of correct compression depth (80.6 ± 38.5%) – were achieved at knee height. Working heights of 15 and 20 cm above knee height yielded significantly lower compression depth results than at knee height. In Part 2, no significant differences in chest compression depth were observed between the three evaluated positions.

**Conclusion:**

Setting the patient’s bed or stretcher to the rescuers’ knee height enables high-quality chest compressions to be delivered while standing. The working height should be adjusted accordingly, with great emphasis placed on avoiding heights that are too high. The findings suggest that the performance of chest compressions while standing may warrant greater attention in future resuscitation guidelines.

## Introduction

When healthcare personnel perform cardiopulmonary resuscitation (CPR) in hospitals, patients are typically lying on a bed or stretcher. Therefore, in emergency departments, intensive care units and operating theatres in particular, chest compressions are usually delivered while the healthcare personnel are standing. To ensure a structured response and maximise the chances of return of spontaneous circulation (ROSC), healthcare personnel in Europe adhere to the recently revised Adult Advanced Life Support (ALS) guidelines issued by the European Resuscitation Council (ERC).^1^

According to the Adult Basic Life Support (BLS) guidelines, high-quality chest compressions (CC) in adults are achieved with a compression depth of 5–6 cm, a rate of 100–120 compressions per minute, and full recoil of the chest after each compression. The guidelines also emphasise that the effectiveness of CC depends on hand positioning, recommending that rescuers keep their arms straight and position their shoulders vertically above the patient's chest. However, these guidelines predominantly address CC delivered from a kneeling position on the floor and provide no specific recommendations for performing chest compressions in a standing position.^2^

Previous studies have demonstrated that rescuer positioning and patient bed height can influence CC quality during CPR.^3^, ^4^, ^5^, ^6^, ^7^, ^8^, ^9^, ^10^, ^11^, ^12^, ^13^, ^14^ In particular, adjustments of working height and the use of step stools have been associated with improvements in compression depth and rescuer ergonomics when performing standing chest compressions.^6^, ^12^, ^15^, ^16^ Existing studies have generally focused on specific equipment configurations or predefined bed heights, whereas the American Heart Association (AHA) recommends positioning the patient’s torso at approximately knee height whenever possible.^17^ To our knowledge, however, no clearly operationalised and clinically applicable adjustment method has been described.

We hypothesised that the height of the stretcher or bed would be a decisive factor in maintaining the correct posture for performing high-quality chest compressions. The aim of this study was to identify a favourable working height for standing chest compressions, to evaluate its performance in comparison with clinically relevant reference conditions and consequently to develop a simple approach for height adjustment.

## Material and methods

### Study design

The study was carried out in two parts. The first part (Approach Development) was designed to determine a working height at which participants achieved the highest percentage of guideline-compliant compression depth, rate, and chest recoil. Based on these results, a simple approach should be developed to help providers identify a favourable working height for CC.

The second part of the study aimed to investigate whether CC performed using this approach were significantly lower in quality than those performed while kneeling on the floor. As setting stretchers to their lowest height appears to be a pragmatic approach in emergency situations, this setting was evaluated additionally.

An a priori sample size calculation was performed for a paired within-subject comparison. Assuming that an optimal working height would result in approximately 80% guideline-compliant compressions and that a clinically relevant difference of 30 percentage points could be detected between height conditions, a minimum sample size of four participants was estimated (*α* = 0.05, power = 0.80). To account for potential dropouts and greater-than-anticipated variability, ten participants were included.

For Part 2, a repeated-measures comparison of three clinically relevant CPR conditions was planned. Owing to the limited availability of prior data for a precise effect-size estimate, the sample size was increased to 20 participants to account for potential variability between conditions.

A total of 30 participants were included in the study, no data sets were excluded. Written consent to participate was obtained from each person in advance. The study protocol was approved by the Ethics Committee of the Medical Faculty of LMU under protocol number 21-0918. The study was conducted over several weeks in June and July 2022 (Approach Development), and November and December 2022 (Evaluation). It took place in the rooms of the Human Simulation Centre (HSC) at the Institut für Notfallmedizin und Medizinmanagement (INM) at the LMU University Hospital.

### Participants

#### Part 1 – Approach development

Ten Emergency Medical Services (EMS) workers from Upper Bavaria and the Munich Fire Department were invited to participate in our study, which involved training and testing their skills in performing chest compressions. All participants were certified as BLS or ALS providers as part of their professional qualifications.

#### Part 2 – Evaluation

Participants in Part 2 were medical students who completed a three-day resuscitation training course (BLS and ALS) at the INM as part of their emergency medical training.

### Study protocol

#### Part 1 – Approach development

We placed a Resusci Anne QCPR AED manikin (Laerdal), equipped with a SimPad Plus (Laerdal), on an emergency stretcher (Trauma Stretcher Model P8000, Hillrom) with a 7.5 cm mattress. In accordance with the ERC guidelines from 2021 and, more recently, 2025, we did not place a backboard beneath the manikin.^2^, ^18^ Bed height was defined as the height of the surface on which the manikin was lying.

The quality of CC was evaluated at seven different table heights. Assigned table heights were participants' knee height, defined as the base of the patella, as well as 10, 15 and 20 cm above (+) and below (−) knee height. In some cases, achieving the designated height setting required the use of 13- or 22-cm-high metal step stools, which are commonly used in German hospitals.

The order of the different height settings for each participant was randomised in advance. For each run, participants performed continuous CC for two minutes and did not receive any feedback on CC quality. Each participant was required to rest for at least six minutes between rounds of chest compressions to minimise the risk of deterioration in quality due to fatigue.^19^ After each run, participants rated the working height on a 5-point Likert scale (1 = very good, 5 = poor). Between runs, they remained in a separate waiting room, where anthropometric measurements (height, arm length, leg length, patella height and weight) and a questionnaire were completed, without observing other participants' CC.

#### Part 2 – Evaluation

In Part 2, we compared the quality of CC while standing at knee level with those performed while kneeling on the ground and with the bed set to its lowest possible height. The order of these settings was also randomised in advance. Step stools were used as well to achieve the desired working height.

After eight participants, the emergency stretcher model was changed from Stryker Prime Series Stretcher (5th Wheel Stretcher) to Hillrom Trauma Stretcher due to a necessary change of location within the INM premises. The lowest possible height settings of the stretchers, including a mattress, were 60.7 cm (Stryker) and 60.2 cm (Hillrom).

### Data collection and analysis

All personal data was collected in a pseudonymised form. The participants' measurements were taken using a standardised anthropometer and body scale. Further parameters, such as gender, age and professional experience were collected via questionnaire.

The following outcome measures were analysed: mean compression depth (MCD), mean compression rate (MCR), the percentage of compressions with guideline-compliant depth (5–6 cm), the percentage of compressions with guideline-compliant rate (100–120 compressions·min⁻^1^), and the percentage of compressions with complete chest recoil.^18^ The manikin automatically recorded compression data, which was extracted using the associated SimPad Skill Reporting Software (version 8.7.0.3). Statistical analysis was performed using IBM SPSS Statistics (version 29.0.0.0). Data are presented as mean ± standard deviation (SD) unless otherwise stated. For Part 1, repeated-measures analysis of variance (rmANOVA) was used to compare outcomes across the seven working-height conditions. Normality was assessed using the Shapiro–Wilk test, and sphericity was evaluated using Mauchly’s test. Whenever the assumption of sphericity was violated, Greenhouse–Geisser corrections were applied. Non-normally distributed percentage data were analysed using Friedman test, followed by Bonferroni-adjusted Wilcoxon signed-rank test (Part 1: six comparisons; Part 2: three comparisons). Associations between anthropometric characteristics and CPR performance outcomes were assessed using Spearman’s rank correlation coefficient (*ρ*). Statistical significance was set at *p* < 0.05.

## Results

### Part 1 – Approach development

#### Participants' characteristics

Of the ten participants included in the first part of the study, four were female (40%), with an average age of 25.2 ± 4.0 years (see Table 1). Their professional experience ranged from one to two years to over six years.

**Table 1:** Characteristics of participants in Part 1 and 2, data are presented as mean ± SD or absolute numbers (percentage), EMS: Emergency Medical Service, EMT: Emergency Medical Technician.

		**Approach development**	**Evaluation**
		**(*N* = 10)**	**(*N* = 20)**
Gender	Female	4 (40%)	12(60%)
	Male	6 (60%)	8(40%)
Age [years]		25.2 ± 4.0	24.3 ± 3.1
Body height [cm]		179.0 ± 9.4	172.6 ± 8.6
Arm length [cm]		59.4 ± 4.8	53.1 ± 3.8
Leg length [cm]		102.6 ± 6.3	98.4 ± 5.7
Base of patella [cm]		54.3 ± 3.5	52.0 ± 2.8
Body weight [kg]		76.8 ± 11.4	68.5 ± 12.5
Occupational field	EMS	7 (70%)	0 (0%)
	Fire Department	2 (20%)	0 (0%)
	Medical school	1 (10%)	20 (100%)
Professional qualification	EMT	4 (40%)	1 (5%)
	Paramedic	4 (40%)	0 (0%)
	Nurse	1 (10%)	1 (5%)
	Student paramedic	1 (10%)	0 (0%)
	Physiotherapist	0 (0%)	1 (5%)
	First Responder	0 (0%)	1 (5%)
Professional experience	0 years	0 (0%)	20 (100%)
	1–2 years	1 (10%)	0 (0%)
	2–6 years	5 (50%)	0 (0%)
	6–10 years	3 (30%)	0 (0%)
	>10 years	1 (10%)	0 (0%)

#### Chest compression depth

**Fig. 1:**
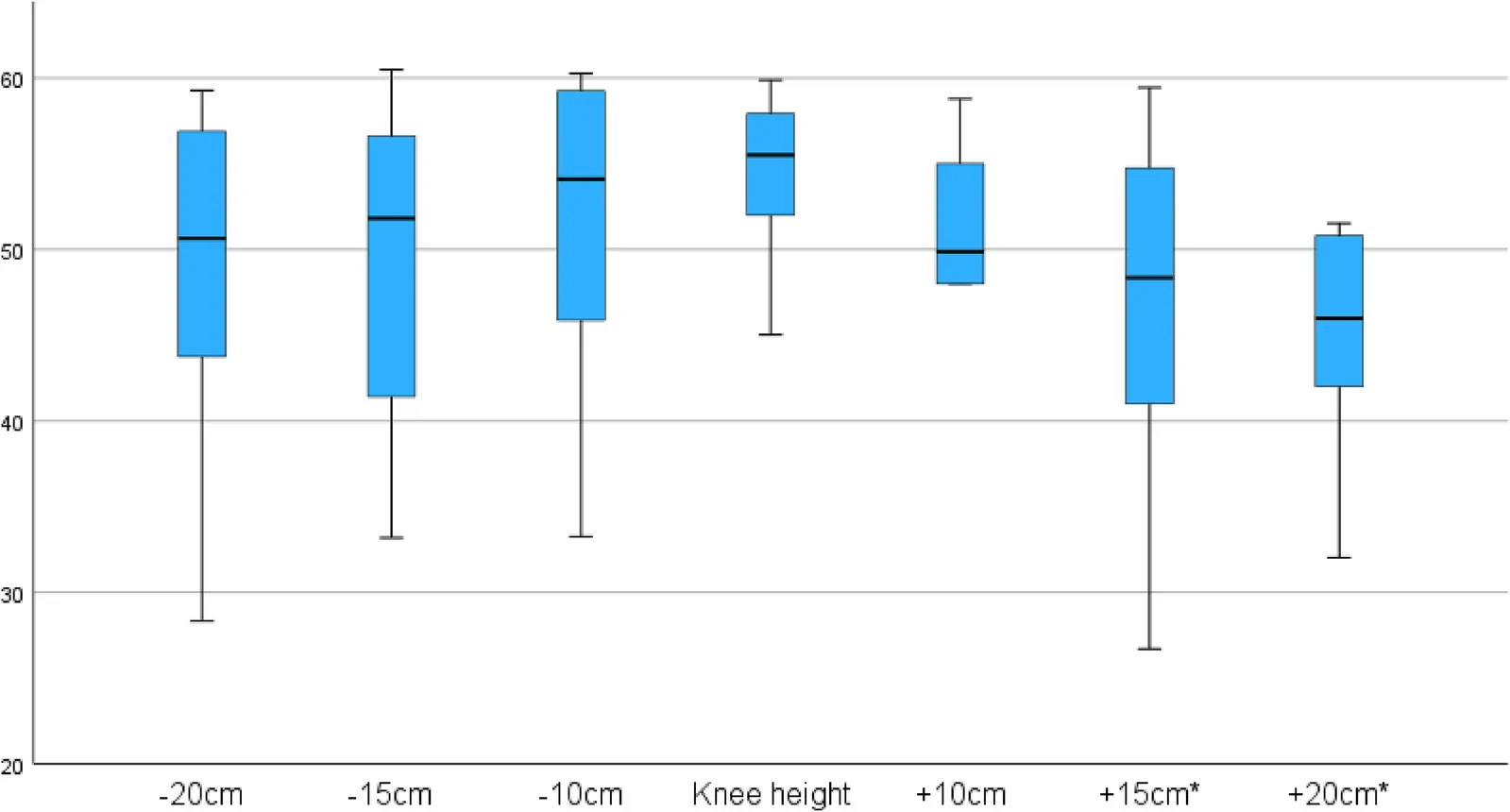
Mean chest compression depth in Part 1, *Y*-axis: compression depth in mm, *X*-axis: setting of the stretcher in relation to the rescuer's knee height; *significantly lower compression depth compared to knee height (*p* < 0.05).

Working height significantly affected mean compression depth (F (6, 54) = 7.64, *p* < 0.001, *η*_p_^2^ = 0.459). MCD and percentage of correct compression depth were highest at knee height (52.7 ± 7.9 mm and 80.6 ± 38.5%). Pairwise comparisons with Bonferroni correction demonstrated significantly greater values at knee height compared with working heights of +15 cm (MD = 5.6, 95%-CI [1.34–9.84], *p* = 0.008) and +20 cm (MD = 7.5, 95%-CI [1.89–13.06], *p* = 0.007). No significant differences were observed between knee height, +10 cm, and the lower working heights. For correct depth percentage, Friedman test confirmed a significant effect of working height (*χ*^2^(6) = 27.95, *p* < 0.001), with only the comparison between knee height and +20 cm remaining significant after Bonferroni adjustment (*p* = 0.048) (see Table 2 and Fig. 1).

**Table 2:** Data are presented as mean ± SD.

		**MCD [mm]**	**Depth compliance [%]**	**MCR [/min]**	**Rate compliance [%]**	**Release compliance [%]**
Part 1: Approach development (*N* = 10)	−20 cm	49.2 ± 9.5	56.9 ± 46.5	118 ± 7	60.3 ± 44.9	97.3 ± 7.8
	−15 cm	49.1 ± 9.0	57.6 ± 49.5	115 ± 6	78.8 ± 32.5	99.6 ± 1.3
	−10 cm	51.6 ± 8.6	62.9 ± 47.9	113 ± 7	77.6 ± 40.3	99.4 ± 1.9
	Knee height	52.7 ± 7.9	80.6 ± 38.5	114 ± 7	81.0 ± 30.5	97.1 ± 5.8
	+10 cm	49.1 ± 8.3	62.3 ± 39.9	112 ± 6	89.0 ± 21.1	96.4 ± 9.5
	+15 cm	47.1* ± 9.6	42.0 ± 43.7	114 ± 9	67.3 ± 33.4	93.8 ± 13.1
	+20 cm	45.2* ± 6.5	34.9* ± 37.0	112 ± 11	55.5 ± 36.3	98.9 ± 2.4

Part 2: Evaluation (*N* = 10)	Knee height	52.8 ± 6.9	70.5 ± 39.7	111 ± 10	68.7 ± 38.9	79.4 ± 29.8
	Floor	54.7 ± 5.6	82.9 ± 28.7	115 ± 7	66.8 ± 29.9	87.0 ± 26.6
	Lowest setting	53.0 ± 6.0	72.5 ± 36.6	109 ± 11	65.9 ± 43.1	86.5 ± 25.7

MCD: Mean Compression Depth, MCR: Mean Compression Rate.

* Significantly lower compression depth compared to knee height (*p* < 0.05).

#### Chest compression rate and complete chest recoil

RmANOVA with Greenhouse-Geisser correction showed no significant differences in mean compression rates (*F*(2.62, 23.58) = 2.099, *p* = 0.134, *η*_p_^2^ = 0.189). Similarly, Friedman test showed no significant differences in the percentage of complete chest recoil (*χ*^2^(5) = 7.11, *p* = 0.213) (see Table 2).

#### Anthropometric measurements

Exploratory analyses revealed no statistically significant associations between the proportion of guideline-compliant CC at knee height and anthropometric measurements. Moderate positive correlations were observed between MCD and several anthropometric variables, although only body weight reached statistical significance (*ρ* = 0.665, *p* = 0.036) (see Table 3).

**Table 3:** Correlation between anthropometric data and compression depth; *ρ*: Spearman's correlation coefficient, *p*-value < 0.05 is considered statistically significant.

	**Approach development (*N* = 10)**				**Evaluation (*N* = 20)**			
	**Mean compression depth**		**Depth compliance**		**Mean compression depth**		**Depth compliance**	
	***ρ***	**(*p*)**	***ρ***	**(*p*)**	***ρ***	**(*p*)**	***ρ***	**(*p*)**
Height	0.58	0.08	0.50	0.15	0.54	0.02	0.47	0.03
Arm length	0.52	0.12	0.37	0.29	0.17	0.17	0.12	0.63
Leg length	0.47	0.17	0.23	0.53	0.58	0.01	0.51	0.02
Patella height	0.58	0.08	0.42	0.22	0.58	0.01	0.53	0.02
Weight	0.67	0.04	0.45	0.19	0.65	<0.01	0.61	<0.01

#### Favourability of different height settings

In Part 1, knee height received the most favourable subjective rating. Overall, subjective ratings were more favourable at knee height and lower working heights, whereas ratings became less favourable at the higher working heights, particularly at +15 and +20 cm (see Table 4).

**Table 4:** Perception of table height for Part 1.

		**−20 cm**	**−15 cm**	**−10 cm**	**Knee height**	**+10 cm**	**+15 cm**	**+20 cm**
Gender	Female	3.3	2.5	2.3	1.0	2.3	3.8	4.8
	Male	2.5	2.3	2.2	1.5	2.7	4.5	5.0
	Total	2.8	2.4	2.2	1.3	2.5	4.2	4.9

Rating: 1 = very good, 2 = good, 3 = satisfactory, 4 = sufficient, 5 = poor.

### Part 2 – Evaluation

#### Participants' characteristics

A total of 20 medical students, with an average age of 24.3 ± 3.1 years, took part in the second part of our study. Of these, 12 (60%) were female. Four of the participants had completed training in nursing, EMS or physiotherapy.

#### Chest compression depth

**Fig. 2:**
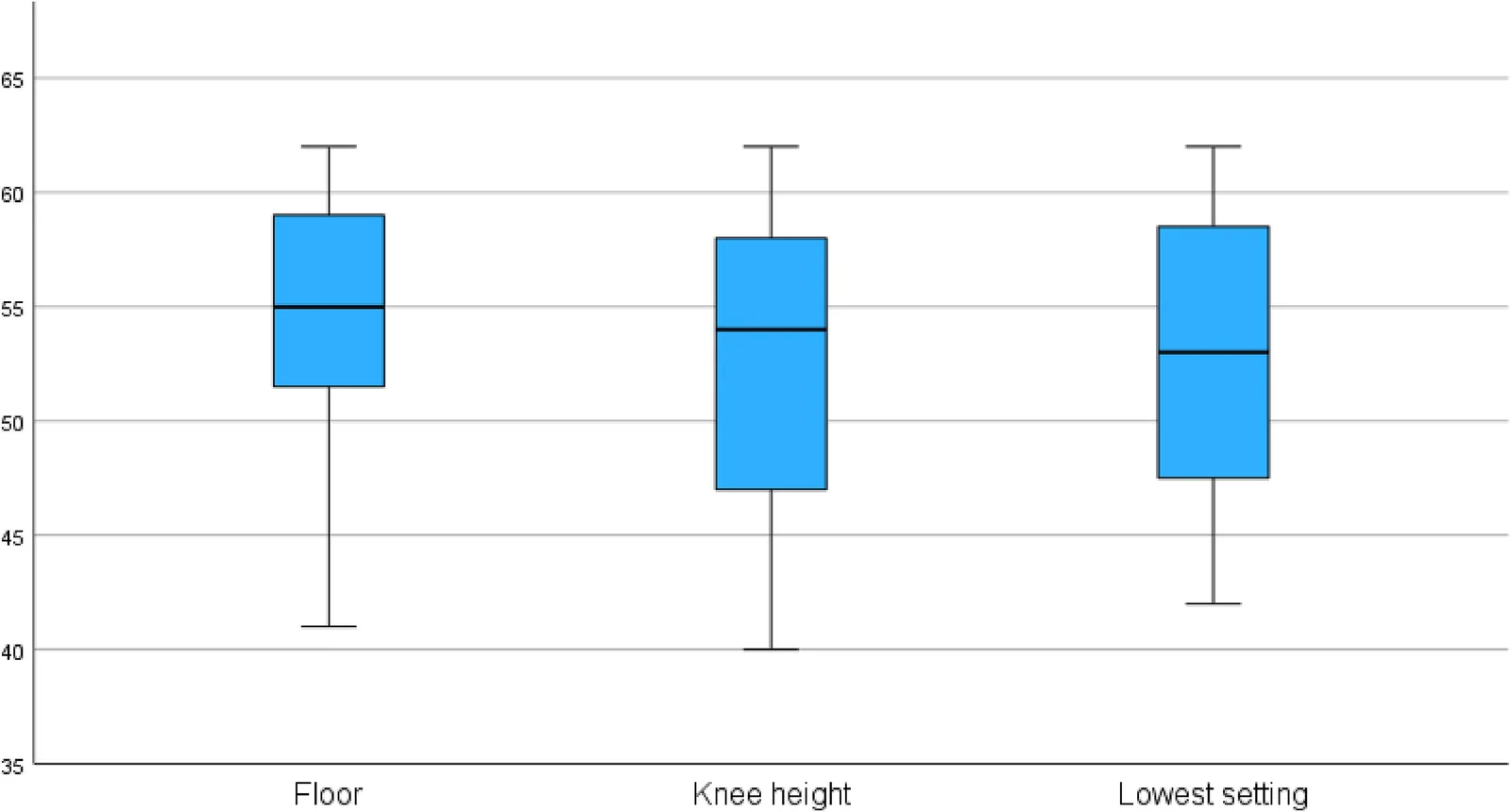
**Chest compression depth in Part 2, *Y*-axis: mean compression depth in mm, *X*-axis: respective working height**.

In Part 2, there were no significant differences between groups observed (MCD: F (2;38) = 1.724, *p* = 0.192, *η*_p_^2^ = 0.083; and % depth compliance: *χ*^2^(2) = 4.073, *p* = 0.131) (see Fig. 2 and Table 2).

#### Chest compression rate and complete chest recoil

RmANOVA with Bonferroni-corrected pairwise comparison revealed a significant difference in MCR between kneeling on the floor and standing with the stretcher set to its lowest possible height (MD = 5.83, 95%-CI [1.39–10.27], *p* = 0.008). No statistically significant differences were found between groups regarding percentage of complete chest recoil (*χ*^2^(2) = 0.571, *p* = 0.751) (see Table 2).

#### Anthropometric measurements

Moderate positive correlations were observed between mean compression depth at knee height and several anthropometric measures, including body height, leg length, patella height, and body weight. The strongest association was found for body weight (Spearman's *ρ* = 0.611, *p* = 0.004) (see Table 3).

#### Favourability of different height settings

In Part 2, delivering chest compressions on the floor was rated best, whereas working at knee height and the lowest stretcher height was perceived as 'satisfactory' (2.7 and 3.2), respectively (see Table 5).

**Table 5:** Perception of table height for Part 2.

		**Knee height**	**Floor**	**Lowest setting**
Gender	Female	2.6	1.6	3.3
	Male	2.8	1.9	3.1
	Total	2.7	1.7	3.2

Rating: 1 = very good, 2 = good, 3 = satisfactory, 4 = sufficient, 5 = poor.

## Discussion

The current ERC guidelines do not provide recommendations on the posture of the rescuer or adequate working height for performing chest compressions while standing.^1^, ^2^ The aim of this study was to conduct and evaluate a simple approach for performing or teaching CPR on a patient lying on a bed or stretcher.

A direct comparison of seven different height settings revealed that setting the lying surface to the rescuer's knee height enabled high-quality, guideline-compliant chest compressions, achieving an average compression depth of 52.7 mm. Participants in Part 1 also found this to be the most comfortable working height. Some authors have examined CC quality at knee height while standing alone and achieved results consistent with the applicable guidelines.^7^, ^8^, ^12^, ^20^ Lewinsohn et al. set the patient's torso at the centre of the rescuer's thigh as a benchmark. Assuming that this corresponds to a bed height set to the rescuer's knee height, as Ho et al. also assume, the results here also showed high-quality chest compressions.^7^, ^8^ However, some authors observed poor CC quality when delivering CC at knee height.^4^, ^5^, ^21^, ^22^ It should be noted that the control groups in these studies also delivered CC well below or, in some cases, above the compression depth recommended by the guidelines.

According to our study, adjusting the height to 10 cm below knee height also enables high-quality CC. Although MCD at 10 cm above knee height is 49.1 mm (slightly below guideline's recommendations), no statistically significant difference was found between knee height and +10 cm. Nevertheless, both heights showed a lower proportion of correctly deep compressions (approximately 63%) than knee height (81%); however, these differences were not statistically significant. Accordingly, other studies have also shown satisfactory results when working height was set at 60 and 63 cm, respectively, which, in our case, corresponds to approximately 10 cm above the patella of the participants and corresponds to setting the stretcher to its lowest setting.^14^, ^23^ Presumably, the most common difficulty when performing standing chest compressions is the working height being set too high. In our study, as well as in others, compression depth and force steadily decreased with an increasing working height.^5^, ^8^, ^24^ Accordingly, our results show that setting the bed or stretcher too low also leads to a deterioration in CC quality. Furthermore, participants in our study clearly preferred working at knee height or slightly below in comparison to higher or lower settings.

We believe that the knee-height position creates optimal conditions because – just as when kneeling on the floor – it allows the shoulders to be kept directly above the compression point. This is not only recommended in the guidelines, but also appears sensible from an ergonomic point of view.^2^ Other studies have also attributed this effect to improved compression depth and higher-quality chest compressions.^3^, ^9^, ^12^, ^13^, ^16^, ^25^ Although this was not investigated in the present study, it appears that human factors and teamwork during CPR play a particularly important role in this regard, as it is relatively easy to judge whether rescuers are standing in an ergonomically favourable position and whether their shoulders are positioned vertically above the compression point.

Although our study participants were relatively tall, it is evident that the mean height of their patellas was still below the lowest height setting of the stretchers used (54 cm and 52 cm vs. 60 cm stretcher height). Although the sample size was small, this suggests that standard hospital stretchers, even at their lowest setting, might be higher than the average knee height of staff. One possible solution would be to use step stools to implement this approach in routine clinical practice. These are already used as standard in operating theatres and numerous studies have shown that they lead to better compression depths. This appears to be due to improved ergonomics for rescuers when delivering chest compressions.^3^, ^6^, ^9^, ^11^, ^12^, ^13^, ^15^, ^16^, ^25^, ^26^

The performance of the knee-height position was subsequently evaluated by comparison with kneeling CPR on the floor and the stretcher set to its lowest height. No statistically significant differences in chest compression quality were observed between conditions, suggesting that high-quality CC can be delivered while standing when the bed or stretcher is positioned at knee height, as well as set to the lowest height. These findings are in line with results shown by Charungwatthana et al., Nicolau et al., and Oh et al.^4^, ^12^, ^20^ Contrary results were primarily obtained in studies in which the table height was not adjusted for the participants, or in which the quality of chest compressions was generally poor.^6^, ^21^, ^26^, ^27^

Although statistically significant differences in compression frequency and chest recoil were observed at different height settings,^14^, ^23^ our study found that these remained unaffected when performed by experienced rescuers, as have other studies.^3^, ^10^, ^12^, ^16^, ^20^, ^26^, ^28^ According to the AHA guidelines, kneeling on the bed is an alternative method of performing CC on a stretcher or bed.^17^, ^26^, ^28^ However, based on further studies, we believe that kneeling on the bed results in poorer quality CC and is often unsafe due to the narrow lying surfaces in emergency rooms.^12^, ^27^, ^29^ If a backboard is used, it can be dangerous or even impossible to kneel next to a patient in bed. In contrast, step stools are routinely used in many clinical environments and may offer a practical means of achieving a more favourable working height. However, the safety implications of step-stool use during resuscitation were not specifically investigated in the present study and need further evaluation.

Another aspect concerns real-time feedback devices. As this study deliberately did not include feedback on the quality of chest compressions, it remains unknown whether, and to what extent, the use of such aids would have influenced the results. Although it is possible that direct feedback could enable better chest compressions to be performed, regardless of the working height, an optimised working height could nevertheless offer advantages from an ergonomic perspective.

Based on the present findings, using the rescuer's patella as a simple anatomical reference point for adjusting working height appears to be a practical approach for delivering high-quality chest compressions while standing. These findings may have practical implications for CPR instructors teaching standing chest compressions and for healthcare professionals during real-world resuscitation efforts. Further studies involving larger samples, experienced medical personnel and different clinical environments are needed to confirm the generalizability of these findings and the relevance of performing CPR in a standing position may warrant consideration in future guideline development.

## Limitations

This study has several limitations. Firstly, the use of a manikin does not measure actual cardiac output and we can therefore only assume that applying our approach enables the best possible patient outcome. Nevertheless, as these quality parameters are recognised as valuable indicators of the quality of chest compressions in BLS and ALS training, we believe that our findings can significantly contribute to optimising the delivery of chest compressions.

Secondly, the sample size in Part 1 was relatively small and the study should therefore be regarded as exploratory. Although the principal findings were confirmed by additional non-parametric analyses, the study may have been underpowered to detect smaller differences between individual height conditions. Furthermore, the individual standard deviations are relatively large, which could cast doubt on the generalisability of the results. Nevertheless, the study aimed to identify differences in CPR quality at different working heights, rather than evaluating individual performance. Although there was substantial variability between participants, the overall trend across the investigated height conditions was clearly recognisable.

Thirdly, the need to adjust the stretcher height in relation to the participants' knee height did not allow for blinding the participants for different working conditions. Nevertheless, randomisation minimised the risk of poor results on the more extreme height settings due to physical exhaustion or anticipation of the subsequent height setting.

Furthermore, all measurements were performed without the use of a backboard, reflecting current resuscitation guideline recommendations. Although this ensured identical testing conditions across all height settings, mattress compressibility may differ between clinical environments. Consequently, the transferability of the proposed knee-height adjustment approach to other bed, stretcher, or mattress systems should be interpreted with caution and warrants further investigation.

Lastly, the Evaluation Part was conducted in a cohort of medical students who had recently completed structured CPR training. Although this ensured familiarity with current resuscitation guidelines, the findings may not be fully generalisable to experienced healthcare professionals or real-world in-hospital cardiac arrest scenarios. Furthermore, the proportion of compressions with complete recoil was lower than expected. While this may reflect the limited practical experience of the participants, MCD remained within ERC recommendations. Consequently, the results should be interpreted as exploratory and there remains a need for studies including larger clinical populations.

## Conclusion

This study suggests that adjusting the patient's bed or stretcher to approximately the rescuer's knee height may facilitate the delivery of high-quality chest compressions while standing. Under the conditions investigated, knee height was associated with the greatest mean compression depth and the highest proportion of guideline-compliant compressions. Also, the findings suggest that a tolerance of approximately 10 cm above and below knee height might be acceptable to enable guideline-compliant chest compressions. The findings may be helpful for healthcare providers performing CPR while standing, however, further studies are necessary to confirm the generalizability of these findings.

## CRediT authorship contribution statement

**Alisha Izadi:** Writing – original draft, Project administration, Methodology, Investigation, Formal analysis, Data curation, Conceptualization. **Selina Kim:** Writing – review & editing, Validation, Methodology, Investigation, Formal analysis, Conceptualization. **Marc Lazarovici:** Writing – review & editing, Resources, Project administration, Methodology, Investigation, Conceptualization. **Stephan Prückner:** Writing – review & editing, Supervision, Resources, Investigation, Funding acquisition. **Heiko Trentzsch:** Writing – review & editing, Validation, Supervision, Project administration, Methodology, Investigation, Formal analysis, Conceptualization.

## Declaration of competing interest

The authors declare that they have no known competing financial interests or personal relationships that could have appeared to influence the work reported in this paper.
